# Enhancing antioxidant capacity via NRF2 pathway activation to mitigate heat stress‐induced oxidative damage in bovine granulosa cells, oocytes, and embryos

**DOI:** 10.3389/fcell.2026.1777760

**Published:** 2026-02-12

**Authors:** Ghyslaine G. Ramírez, Ahmed Gad, Nico G. Menjivar, Scott Burlingham, Ming-Hao Cheng, Thomas W. Chen, Soham Ghosh, Dawit Tesfaye

**Affiliations:** 1 Animal Reproduction and Biotechnology Laboratory (ARBL), Department of Biomedical Sciences, College of Veterinary Medicine and Biomedical Sciences, Colorado State University, Fort Collins, CO, United States; 2 Animal Reproduction and Biotechnology Laboratory (ARBL), Department of Clinical Sciences, College of Veterinary Medicine and Biomedical Sciences, Colorado State University, Fort Collins, CO, United States; 3 Department of Animal Production, Faculty of Agriculture, Cairo University, Giza, Egypt; 4 Stanford Children’s Health. Lucile Packard Children’s Hospital, Stanford Medicine, Fertility and Reproductive Health, Sunnyvale, CA, United States; 5 Cellular Engineering and Mechanobiology Laboratory (CEML), Translational Medicine Institute (TMI), Department of Mechanical Engineering, Colorado State University, Fort Collins, CO, United States; 6 Department of Electrical & Computer Engineering, Colorado State University, Fort Collins, CO, United States; 7 School of Biomedical Engineering, Colorado State University, Fort Collins, CO, United States

**Keywords:** heat stress, NRF2 pathway, antioxidants, embryonic development, oocyte maturation, granulosa cells, quercetin, sulforaphane

## Abstract

Global warming-induced thermal stress is an escalating threat to livestock fertility, perturbing ovarian function, oocyte maturation, and preimplantation embryo development through excessive accumulation of reactive oxygen species (ROS), which drive follicular oxidative damage. Although embryo transfer technologies offer a practical abatement strategy for mitigating such implications caused by HS, *in vitro* systems lack the endogenous antioxidant defenses present *in vivo*, leaving follicular cells, gametes, and embryos particularly vulnerable. Here, we aimed to investigate whether antioxidants, including quercetin (QUE), carnosol (CAR), and sulforaphane (SFN), mitigate HS-induced follicular oxidative damage in bovine granulosa cells (GCs), oocytes, and embryos. For this, antioxidant supplementation, either individually or in combination, was performed during *in vitro* GC culture and oocyte maturation under normothermic (NT) or HS conditions. Across all models, QUE and SFN supplementation activated nuclear NRF2, reduced ROS accumulation, and restored mitochondrial function and apoptosis levels under conditions of HS. In oocytes exposed to thermal stress, QUE and SFN supplementation also led to increased blastocyst rates and total cell numbers. Single-embryo metabolic profiling revealed reduced oxygen consumption (OCR) and extracellular acidification (ECAR) rates in blastocysts derived from antioxidant-treated oocytes, indicative of enhanced metabolic efficiency. Moreover, quantitative analysis of recently defined embryo competence-associated genes demonstrated a restoration of the embryo competence index (ECI) following QUE and SFN supplementation. In conclusion, antioxidant supplementation during GC culture and oocyte maturation alleviates HS-induced reproductive dysfunction by restoring redox homeostasis, preserving metabolic efficiency, and re-establishing embryo competence, thereby providing a mechanistically grounded strategy to mitigate climate-driven fertility decline in cattle.

## 1 Introduction

Heat stress (HS), defined as the inability to adequately dissipate body heat, exerts well-documented detrimental effects on cattle reproductive physiology and productivity (Khan et al., 2023; Lovarelli et al., 2024; Wolfenson et al., 2000; Wolfenson and Roth, 2019). The impact of HS on female fertility is profound and multifaceted. At the molecular level, within the ovarian follicular microenvironment, HS leads to the accumulation of reactive oxygen species (ROS), triggering oxidative stress, endoplasmic reticulum (ER) stress, mitochondrial dysfunction, and apoptosis (Amaral et al., 2020). Specifically in the ovary, ROS accumulation has been shown to deteriorate oocyte quality, largely by inducing GC apoptosis and accelerating corpus luteum degeneration (Liu et al., 2020; Zhang et al., 2023). As oocyte maturation occurs synchronously with follicular development, and oocyte competence depends on the bidirectional communication with the surrounding GCs, oocytes are therefore highly vulnerable to ROS. Cumulus cells (CCs) deliver mRNA cargo to the oocyte through trans-zonal projections (TZP), which contribute to the oocyte mRNA reserve (Macaulay et al., 2016). These early oogenic events are crucial for the embryo’s developmental potential. Moreover, the long-term impact of HS stress during oocyte maturation on embryonic developmental potential and molecular architecture, mediated in part through epigenetic modifications, has been previously demonstrated (Amaral et al., 2020; Bie et al., 2017; Kono et al., 1996).

In response to thermal stress, GCs, oocytes, and embryos rely on the cascade of protective antioxidant systems to maintain cellular homeostasis, including the induction of heat shock proteins (HSPs) and the unfolded protein response (UPR) (Marei and Leroy, 2022). A key component of the antioxidant defense machinery is the transcription factor Nuclear factor erythroid 2-related factor 2 (NRF2), which regulates enzymes that mitigate oxidative damage in GCs, oocytes, and preimplantation embryos (Amin et al., 2014; Feng et al., 2023; Khadrawy et al., 2020). As a master regulator of cellular redox homeostasis, NRF2 controls the expression of more than 100 oxidative stress-associated genes, mitigating cellular damage caused by pro-oxidants (Chen, 2022; Lu et al., 2016; Thiruvengadam et al., 2021). Under physiological conditions, NRF2 is tightly regulated by Kelch-like ECH-associated protein 1 (KEAP1), facilitating NRF2’s continuous ubiquitination and degradation. We have previously shown that failure of embryos to activate their NRF2-mediated oxidative stress response can lead to accumulated ROS, reduced mitochondrial function, and a subsequent reduction in developmental competence (Aglan et al., 2020; Amin et al., 2014; Khadrawy et al., 2019; Khadrawy et al., 2020; Sohel et al., 2018; Taqi et al., 2021). Pharmacological activation of NRF2 through natural plant compounds has also emerged as a promising strategy (Khadrawy et al., 2019; Taqi et al., 2021). Although the precise molecular interactions of most plant compounds remain incompletely characterized, the binding interactions of quercetin (QUE), carnosol (CAR), and sulforaphane (SFN) with KEAP1 are well established (Hong et al., 2005; Ji et al., 2023; Luo et al., 2022). QUE, a natural antioxidant polyphenol, binds to KEAP1 at Arg483 through hydrogen bonds and also promotes NRF2 phosphorylation through PKC, thereby enhancing NRF2 activity and reducing oxidative stress (Luo et al., 2022). CAR, another natural polyphenol, is reported to form hydrogen bonds with amino acids Serine602 and Glutamine530 and π-π stacking interaction with Tyr572 of KEAP1, activating NRF2 signaling and ameliorating PCOS-related phenotypes in KGN cells (Ji et al., 2023). SFN, abundant in cruciferous vegetables, modifies KEAP1 cysteine residues through a reversible reaction, mimicking ROS-induced KEAP1 modification (Hong et al., 2005). Functional studies demonstrate that SFN induces NRF2 nuclear translocation, increases antioxidant enzyme expression, and rescues GCs from oxidative damage *in vitro* (Sohel et al., 2017). This highlights the need to unveil whether exogenous antioxidant supplementation effectively enhances the antioxidant capacity of GCs, oocytes, and the resulting blastocysts, thereby mitigating the impact of environmental stress. Here, we investigated whether supplementation with QUE, CAR, and SFN during GCs and oocyte culture under normothermic and HS conditions could enhance NRF2-mediated cellular defense mechanisms and improve metabolic and embryonic competence.

## 2 Materials and methods

### 2.1 Ovarian collection

Bovine ovaries were obtained from a local abattoir and transported to the laboratory within 1 h. Ovaries were placed in an insulated container filled with physiological saline solution (0.9% NaCl) at 37 °C. Upon arrival, ovarian samples were immediately washed three times with a warmed 0.9% NaCl solution, followed by a rinse with 70% ethanol.

### 2.2 Granulosa cells (GCs) and cumulus-oocyte-complex (COCs) isolation and culture

Follicular fluid containing GCs and COCs was aspirated from ovarian follicles ranging between 3 and 8 mm in diameter using a vacuum pump (GenX International; Guelph, ON, Canada) calibrated to 50 mmHg. The pumping system was connected to a standard 18-gauge hypodermic needle (Covidien Monoject™; Mansfield, MA, United States) and paired with a sterile 50 mL collection tube (CELLTREAT® Scientific Products; Pepperell, MA, United States). The aspirated follicular fluid was left to settle undisturbed for 10 min, allowing a visible cellular pellet containing COCs to form at the bottom of the tube. The supernatant containing GCs was then carefully transferred to a sterile 15 mL tube (Thermo Fisher Scientific; Waltham, MA, United States) with 3 mL of warm Dulbecco’s Phosphate Buffered Saline (DPBS (1x)) without calcium and magnesium chloride (Sigma-Aldrich; St. Louis, MO, United States).

#### 2.2.1 Antioxidant dilutions and preparation

Quercetin (QUE; Sigma-Aldrich; St Louis, MO, United States), Carnosol (CAR; MedChemExpress; Monmouth Junction, NJ, United States), and Sulforaphane (SFN; Selleck Chemicals; Houston, TX, United States) were prepared at 10 mM stock solution in dimethyl sulfoxide (DMSO; Sigma-Aldrich; St Louis, MO, United States). To ensure consistency across treatments, the final DMSO concentration was standardized and maintained constant for all experimental groups, including the vehicle controls. Working dilutions were freshly prepared immediately before use.

#### 2.2.2 Granulosa cells (GCs) culture, antioxidant supplementation, and heat stress (HS) exposure

The follicular fluid suspension containing GCs was centrifuged at 500 × g for 7 min, after which the GC pellet was collected and the supernatant discarded. The pellet was resuspended in red blood cell (RBC) lysis buffer (Sigma-Aldrich; St Louis, MO, United States) and incubated for 3 min. The reaction was stopped by washing the cells in Dulbecco’s Modified Eagle’s Media/Ham’s Nutrient Mixture F12 (DMEM/F12) (Sigma-Aldrich; St. Louis, MO, United States) supplemented with 10% fetal bovine serum (FBS, Sigma-Aldrich; St. Louis, MO, United States), 1% Penicillin-Streptomycin (Sigma-Aldrich; St. Louis, MO, United States), and 1% Amphotericin B solution (Sigma-Aldrich; St. Louis, MO, United States). The cells were then centrifuged again at 500 × g for 5 min. This process was repeated until a pure pellet was obtained. The supernatant was discarded, and the pellet was washed with DPBS (1x) before being resuspended in the DMEM/F12 media described above. Quantity and viability of GCs were assessed using a hemocytometer (Hausser Scientific, Horsham, PA, United States) and the trypan blue exclusion method (Sigma-Aldrich; St. Louis, MO, United States). Depending on the assay, GCs were seeded at 2 × 10^5^ cells per well in 24-well plates or at 1 × 10^4^ cells per well in 96-well tissue-culture-treated plates (United States Scientific, Inc., Ocala, FL) in DMEM/F12 media as described above.

Bovine GCs were initially cultured for 24 h until reaching subconfluency. Antioxidant supplementation was performed following a brief wash a with DPBS (1x) for QUE (5 µM), CAR (10 µM), SFN (1 µM), a combination of three (MIX), vehicle (DMSO), and an untreated control for 24 h. Subsequently, control groups were maintained under normal temperature (NT, 38.5 °C) for 24 h, while the HS-treated groups were transferred for 8 h to a different incubator programmed at 42 °C and then returned to normal temperature (NT) for the remaining 16 h, a method to mimic daylight conditions, and summing in a 72 h total length of culture. The 42 °C temperature is commonly used *in vitro* to induce a robust and reproducible heat-stress response without causing excessive cell death in bovine GCs (Roth and Hansen, 2004). A schematic overview of the experimental design is shown in (Supplementary Figure S1A).

#### 2.2.3 *In vitro* maturation (IVM), antioxidant supplementation, and heat stress (HS) exposure

COCs were collected from abattoir ovaries as described above. Retrieved COCs were grouped (50 per well) and placed into 4-well culture dishes (Thermo Fisher Scientific, Waltham, MA, United States) containing 1 mL of chemically defined media for *in vitro* maturation of oocytes (IVM) pre-equilibrated at 38.5 °C in 5% CO_2_ (De La Torre-Sanchez et al., 2006). At the time of equilibration, all IVM media were supplemented with 15 ng/mL NIDDK-oFSH-20, 1 μg/mL USDA-LHB-5, 1 μg/mL estradiol 17β, 50 ng/mL epidermal growth factor, and 0.1 mM cysteamine (De La Torre-Sanchez et al., 2006). Based on their ability to enhance nuclear activation of the NRF2 protein in cultured GCs, only QUE and SFN were selected for further evaluation during the oocyte maturation experiment. Consequently, the six experimental groups used during GC culture were reduced to four groups for the oocyte maturation experiments: 5 μM QUE under HS, 1 μM SFN under HS, and vehicle control (DMSO) under both thermoneutral and HS conditions.

*In vitro* oocyte maturation, which lasted 23 ± 1 h, was initiated by incubating all treatment groups under normothermic conditions (NT; 38.5 °C) for the first 8 h. Subsequently, the HS QUE, HS SFN, and HS control groups were transferred to a separate incubator set at 41 °C for the remaining 15 h, whereas the NT control group remained under NT conditions throughout the IVM period. Previous studies have shown that exposure of bovine oocytes to 41 °C during IVM is sufficient to impair developmental competence, supporting its use as a physiologically relevant heat stress (HS) model (Paula-Lopes and Hansen, 2002). A schematic overview of the experimental design is shown in (Supplementary Figure S1B).

### 2.3 *In vitro* fertilization (IVF) and embryo culture

For *in vitro* fertilization (IVF), COCs were transferred (in <20 μL of maturation media) into 4-well culture dishes containing 430 μL of equilibrated CSU chemically defined media for IVF (F-CDM) (De La Torre-Sanchez, Jose Fernando et al., 2006) and maintained at 38.5 °C and 5% CO_2_ in humidified air. Frozen-thawed bull sperm from a single ejaculation were processed to separate viable and motile sperm using a multilayer 45%/90% Percoll® (Sigma-Aldrich; St. Louis, MO, United States) gradient and centrifuged at 800 × g for 20 min. Following this, the supernatant was discarded, and the remaining sperm pellet was washed with 2 mL of CSU chemically defined media for the handling of early embryos (HCDM-1) (De La Torre-Sanchez et al., 2006) and centrifuged at 300 × g for 5 min. Sperm concentration was determined using a hemocytometer, and the suspension was adjusted to 5 × 10^6^ sperm/mL using equilibrated F-CDM. Diluted sperm was then added to COCs at a final concentration of 0.5 × 10^6^ sperm/mL and co-incubated for 18 h at 38.5 °C in 5% CO2 in humidified air. Following fertilization, presumptive zygotes were vortexed for 90 s at maximum speed to remove cumulus cells (CCs). Denuded zygotes were then transferred in groups of 50 to pre-equilibrated 4-well dishes containing 500 μL of CSU chemically defined media for *in vitro* culture of early embryos (CDM-1). Embryos were cultured for 56 h in a mixed gas incubator maintained at 38.5 °C in 5% CO_2_, 5% O_2_, and 90% N_2_. Embryos with ≥4 blastomeres were then transferred in groups of 35 to pre-equilibrated 4-well dishes containing 500 μL of CSU chemically defined media for *in vitro* culture of late embryos (CDM-2). Embryos were cultured for 120 h in a mixed gas incubator maintained at 38.5 °C in 5% CO_2_, 5% O_2_, and 90% N_2_ (De La Torre-Sanchez et al., 2006). Blastocyst developmental rates were evaluated on day 7 (168 h) and day 8 (192 h) post-IVF. A schematic overview of the experimental design is shown in (Supplementary Figure S1C).

### 2.4 Antioxidant dose optimization and cell viability assay for bovine GCs

Bovine GC collection, culture, HS treatment, and antioxidant supplementation were performed as indicated above. Cell viability was assessed using the Cell Counting Kit-8 (CCK-8, Dojindo Molecular Technology, Japan). Doses tested were 1, 5, 10, and 20 μM for QUE, CAR, and SFN under NT and HS conditions. For this, 10 μL of the reagent was added to each well and incubated for 3 h at 38.5 °C in a 5% CO2 incubator. Optical density of the formazan dye was measured at 450 nm using a microplate reader (BioTek Instruments, Germany). Wells containing only media with the antioxidant, but no cells, served as blanks for background correction. All assays were performed in quadruplicate. Based on these results, the highest non-toxic doses were selected: 1, 5, and 10 μM for SFN, QUE, and CAR, and an antioxidant mix was based on these doses (Supplementary Figure S2). Additionally, these doses were used for further experiments.

### 2.5 Active NRF2 binding assay on nuclear extracts from bovine GCs

Nuclear activation of NRF2 in antioxidant-supplemented bovine GC nuclear extracts under NT and HS was assessed using the commercial Trans-AM® NRF2 kit (Active Motif, Carlsbad, CA, United States). Following culture, the NEPER Nuclear and Cytoplasmic Extraction Kit (Thermo Fisher Scientific; Waltham, MA, United States) was employed to separate nuclear and cytoplasmic protein fractions from bovine GCs from four biological replicates. Protein concentrations in the resulting nuclear extract suspensions were measured using the Pierce BCA protein assay kit (Thermo Fisher Scientific; Waltham, MA, United States) following the manufacturer’s instructions. Subsequently, the TransAM® NRF2 ELISA was used to analyze the nuclear protein extracts from individual and combined antioxidant-supplemented groups under NT and HS conditions. Optical density was measured at 450 nm with a reference wavelength of 655 nm using a microplate reader (BioTek Instruments, Germany). Wells containing all reactive agents was used as a blank for background correction.

### 2.6 Annexin V/propidium iodide (PI) staining on bovine GCs

To assess the apoptotic index (early apoptosis + late apoptosis) in bovine GCs, co-staining of annexin V and propidium iodide (PI) was performed using the Cell Meter APC-Annexin V Binding Apoptosis Assay Kit (Biomol International; Plymouth Meeting, PA, United States), according to the manufacturer’s recommendations with minor modifications. GCs were analyzed using the Cytek 4 laser-Aurora™ (Cytek Biosciences; Fremont, CA, United States). Assays were performed in duplicate, and the percentages of different cell populations within the treatment groups (live and healthy, early apoptotic, late apoptotic, necrotic) were recorded for analysis.

### 2.7 Gene expression analysis of antioxidant and stress-related markers in GCs, denuded oocytes, and CCs, and embryo competence-associated genes in blastocysts

The impact of antioxidants on modulating stress response genes in GCs, denuded oocytes, and CCs was investigated using qRT-PCR. Total RNA from GCs was isolated from three replicates using the miRNeasy® mini kit (Qiagen; Hilden, Germany). Moreover, total RNA was extracted from four pooled replicates of denuded oocytes and CCs (50 COCs) and from blastocysts (10 blastocysts) using the RNeasy® plus micro kit (Qiagen; Hilden, Germany). Total RNA sample concentrations and integrity were assessed using a NanoDrop 2000 Spectrophotometer (Thermo Fisher Scientific; Waltham, MA, United States). RNA samples were stored at −80 °C until further use.

Following this, cDNA was synthesized by reverse transcription using oligo(dT)_18_ primers and the First-Strand cDNA Synthesis Kit (Thermo Fisher Scientific; Waltham, MA, United States). For each sample type, cDNA synthesis was performed using the maximum amount of high-quality RNA available per biological replicate, resulting in different RNA input amounts for granulosa cells (33.2 ng), denuded oocytes (22.2 ng), cumulus cells (29.4 ng), and blastocysts (16.8 ng). These differences reflect biological and technical limitations in RNA yield inherent to each sample type, rather than differences in experimental normalization. Gene expression analyses were conducted for each sample type, and relative expression levels were normalized to the geometric mean of the reference genes β-ACTIN and GAPDH. Gene-specific primers were designed using Primer-Blast (https://www.ncbi.nlm.nih.gov/tools/primer-blast/), and the list of primers is indicated in Supplementary Table S1. Formal qRT-PCR analysis was performed using the CFX96 Touch Real-Time PCR Detection System (Bio-Rad; Hercules, CA, United States). The following thermocycling conditions were applied for amplification: initial denaturation at 95 °C for 3 min, followed by 40 cycles of amplification at 95 °C for 15 s, and 60 °C for 60 s. The specificity of the amplification was determined by melting curve analysis generated at the end of each run, and the data were analyzed using the comparative C(T) method (Schmittgen and Livak, 2008).

### 2.8 Proximity ligation assay (PLA) of KEAP1-NRF2 protein-protein interactions in preimplantation embryos

Following the manufacturer’s NaveniFlex PLA protocol (Navinci Diagnostics, Uppsala, Sweden), the embryos were counterstained using two primary antibodies (NRF2 polyclonal antibody, Invitrogen; Carlsbad, CA, United States, unconjugated, rabbit polyclonal antibody, PA138312; KEAP1 (G-2), Santa Cruz Biotechnology; Dallas, TX, United States, mouse monoclonal antibody, sc-365626) and mounted on a poly-L-lysine-coated slide (Newcomer Supply; Middleton, WI, United States) with 5–10 μL of ProLong Diamond Antifade Mountant (Invitrogen; Carlsbad, CA, United States) and a coverslip, allowing them to dry flat (>24 h). PLA/DAPI-labeled embryos were imaged using the LSM980 (Carl Zeiss Microscopy, Jena, Germany) equipped with a 60× oil objective with fitted blue (345/455) and red (596/615) fluorescent filters. For the analysis, fluorescent images were converted to grayscale, puncta counts and total cell counts were obtained using ImageJ software (National Institute of Health, Bethesda, MD, United States). The ratio of PLA puncta count normalized to total cell number from individual blastocysts was subsequently calculated. For each treatment group, 6–11 blastocysts were analyzed.

### 2.9 Mitochondrial function assays on bovine GCs, denuded oocytes, and preimplantation embryos

#### 2.9.1 Intracellular reactive oxygen species (ROS) accumulation assay

The impact of thermal stress on intracellular ROS accumulation was assessed using 10 μM 2′, 7′-Dichlorofluorescein Diacetate (H2DCFDA) (Sigma Aldrich; St. Louis, MO, United States) on GCs, bovine denuded oocytes, and blastocysts. GCs were seeded at 2 × 10^5 cells per well in 24-well plates containing 600 μL of media as described above. Following the culture (washing, antioxidant supplementation, and heat exposure), the media was removed, and cells from both NT and HS treatments were washed twice with DPBS (1x) (Sigma-Aldrich; St. Louis, MO, United States). Cells were then incubated with 10 μM H2DCFDA diluted in 200 μL of non-supplemented F12 culture media for 30 min in a CO2 incubator. After incubation, cells were washed three times using DPBS (1x) (Sigma Aldrich; St. Louis, MO, United States), and immediately imaged using an inverted fluorescence microscope (IX73, Olympus, Tokyo, Japan). Fluorescence intensity was quantified from. jpg images from different biological replicates, taken from two specific locations per well, across four wells per treatment group per replicate (n = 16 images per group). All images were acquired with identical exposure and light settings, avoiding signal saturation, using a green fluorescent filter (excitation 494 nm, emission 518 nm). Fluorescence intensity was analyzed using ImageJ software (National Institute of Health, Bethesda, MD, United States).

ROS accumulation in denuded oocytes (n = 20 per treatment) and blastocysts (n = 14 per treatment) was assessed as described above. Briefly, denuded oocytes and blastocysts from each treatment group were washed twice in phosphate-buffered saline supplemented with 0.1% polyvinylpyrrolidone (DPBS (1x)-PVP) (Sigma Aldrich; St. Louis, MO, United States). Samples were then incubated for 30 min at 38.5 °C in 50 μL drops of 10 μM H2DCFDA dissolved in H-CDMM (for oocytes) or HCDM-2 (for blastocysts). Following incubation, oocytes and blastocysts were washed three times in DPBS (1x) containing PVP (0.1%) and immediately imaged using an inverted fluorescence microscope (IX73, Olympus, Tokyo, Japan). Fluorescence intensity was quantified from. jpg images by manually selecting the oocyte or blastocyst area. All images were acquired with identical exposure and light settings, avoiding signal saturation, using a green fluorescent filter (excitation 494 nm, emission 518 nm). Fluorescence intensity was analyzed using ImageJ software (National Institute of Health, Bethesda, MD, United States).

#### 2.9.2 Mitochondrial Membrane Potential (MMP) δΨm assay

The detrimental effect of heat stress on mitochondrial depolarization was analyzed using the cationic dye 5,5′,6,6′-tetrachloro1,1′,3,3′ tetraethyl-benzimidazolyl-carbocyanine iodide (JC-1, Invitrogen, Carlsbad, CA, United States) on GCs, denuded oocytes, and blastocysts. GCs were seeded at 2 × 10^5 cells per well in 24-well plates containing 600 μL of media as described above. Following the culture (washing, antioxidant supplementation, and heat exposure), the media was removed, and cells from both NT and HS treatments were washed twice with DPBS (1x) (Sigma-Aldrich; St. Louis, MO, United States). Cells were then incubated with 2 μM JC-1 staining diluted in 200 μL media culture for 30 min in a CO2 incubator. After incubation, cells were washed three times using DPBS (1x) (Sigma Aldrich; St. Louis, MO, United States) and immediately imaged using an inverted fluorescence microscope (IX73, Olympus, Tokyo, Japan). Images were captured at two defined locations per well, across four wells per treatment group per replicate (n = 16 total per treatment).

MMP (δΨm) in oocytes (duplicates; a total of 20 oocytes per treatment) and blastocysts (duplicates; a total of 14–15 blastocysts per treatment) was evaluated using the cationic dye 5,5′,6,6′-tetrachloro-1,1′,3,3′-tetraethylbenzimidazolylcarbocyanine iodide (JC-1; Invitrogen, Carlsbad, CA, United States), dissolved in H-CDMM for oocytes and HCDM-2 media for blastocysts, as an indicator of mitochondrial activity, as previously described (Pasquariello et al., 2019). Briefly, denuded mature oocytes and blastocysts from the respective treatment groups were incubated with 2 μM JC-1 for 30 min in the dark at 38.5 °C under their optimal culture conditions. Following incubation, samples were washed three times with DPBS (1×) supplemented with 0.1% PVP and immediately imaged using an inverted laser scanning confocal microscope. Oocytes were imaged using the ZEISS LSM800 equipped with a 20× air objective, while blastocysts were imaged using the ZEISS LSM980 (Carl Zeiss Microscopy, Jena, Germany) equipped with a 60× oil objective.

Fluorescence intensity was quantified from. jpg (for GCs) and. czi (for oocytes and blastocysts) images obtained from different biological replicates. Imaging for J-monomers was conducted using a wavelength of 488 nm for J-monomers and excitation/emission settings at 490/525 nm (FITC filter, green fluorescence), indicative of low MMP. Similarly, images for J-aggregates were carried out using a wavelength of 561 nm and excitation/emission at 596/615 nm (TRITC filter/red fluorescence), indicating high MMP. Fluorescence intensity was analyzed using ImageJ software (National Institute of Health, Bethesda, MD, United States). The ratio of red to green fluorescence was subsequently calculated to determine MMP, expressed as an arbitrary unit.

#### 2.9.3 Preimplantation embryo oxygen consumption rate (OCR) and extracellular acidification rate (ECAR) assays

Metabolic analyses of individual day 7 blastocysts (n = 4–17) were performed using a microchamber with electrochemical-based oxygen and pH sensors, as described (Obeidat Y. et al., 2019; Obeidat Y. M. et al., 2019). Briefly, the microchamber was filled with 150 μL of CSU chemically defined media (CDM-2). Then individual embryos were placed in the middle of each of the six channels containing CDM-2 media. The three-electrode oxygen sensor was connected to a potentiostat (Quadstat EA 164H, eDAQ Inc., Colorado Springs, CO) that applied a voltage to the sensor and monitored the decrease in oxygen-reduction current over time. The applied potential for all amperometric oxygen assays was set to −0.6 V. The pH sensor was connected to a custom-made Ina333 instrumentation amplifier circuit that measured the change in voltage. The starting (room air-saturated) oxygen concentration and pH of the media were measured as baseline values for 2 min and calibrated as previously described in detail (Obeidat Y. et al., 2019).

### 2.10 Statistical analysis

Data were analyzed in GraphPad Prism version 8.4.2 (GraphPad; San Diego, CA, United States). For experiments in which all treatments were tested at both temperatures, a two-way Analysis of Variance (ANOVA) was performed with temperature and treatment as fixed factors, followed by Tukey’s multiple-comparison test. When treatments were applied at only one temperature (HS), data were analyzed using one-way ANOVA, and Tukey’s *post hoc* test was used to compare treatment groups with the corresponding temperature-matched control. Data are presented as the Mean ± SEM of biological replicates. Statistical significance was identified at p ≤ 0.05.

## 3 Results

### 3.1 Dose optimization for antioxidant supplementation in bovine GCs

To determine the optimal antioxidant concentrations, we first evaluated doses of 1, 5, 10, and 20 μM for QUE, CAR, and SFN. As indicated in Supplementary Figure S2, most of the doses tested did not compromise cell viability, confirming their safety and efficacy under conditions of HS. Based on these results, we then selected the highest non-toxic, biologically active concentrations,1 μM SFN, 5 μM QUE, and 10 μM CAR, for all subsequent experiments. In addition, a combinatorial treatment (MIX) was also included to assess potential synergistic effects antioxidants. For all endpoints evaluated, two-way ANOVA revealed a significant temperature × antioxidant treatment interaction (*p < 0.05*), indicating that antioxidant effects were dependent on the presence or absence of thermal stress conditions.

### 3.2 Impact of QUE, CAR, and SFN on the cellular NRF2-mediated oxidative stress response pathway

**FIGURE 1:**
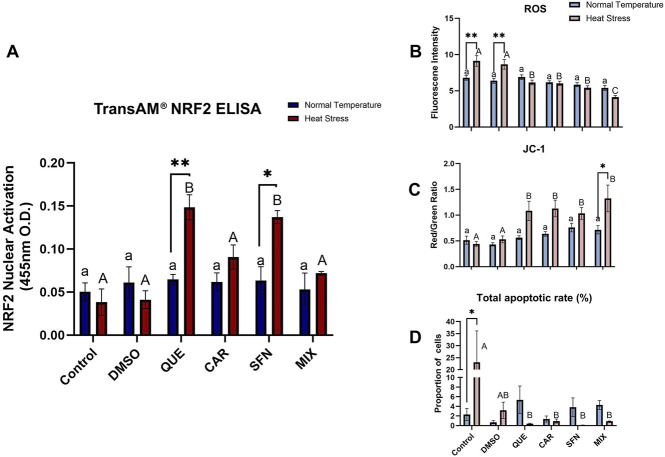
The beneficial effect of antioxidant supplementation on NRF2 protein nuclear expression and cellular homeostasis in bovine GCs. **(A)** TransAM® NRF2 ELISAs were used for nuclear protein extracts from antioxidant-supplemented bovine granulosa cells under Normal Temperature and Heat Stress. Gene expression analysis to quantify selected stress-related genes NRF2, SOD1, HO1, PRDX1, CAT, HSP70, HSP90, GRP78, and GRP94 is shown in Supplementary Figure S3A. Blue bars represent Normal Temperature groups, and red bars represent Heat Stress treatments. **(B)** Reactive Oxygen Species (ROS) accumulation assay 2′,7′- DCFH-DA quantification of the relative fluorescence intensity of Reactive Oxygen Species (ROS) in bovine GCs. **(C)** Quantitative red/green fluorescence intensity ratio using 5,5′,6,6′tetrachloro-1,1′,3,3′tetraethylbenzimidazolylcarbocynanine iodide (JC-1) for the measurement of Mitochondrial Membrane Potential (ΔΨm), an indicator of mitochondrial activity in bovine GCs. Green fluorescence represents JC-1 monomers, whereas red fluorescence represents JC-1 aggregates. **(D)** Annexin V/PI staining was evaluated using flow cytometry to determine the total apoptotic rate, including the proportion of early and late apoptotic cells. Representative panels shown in Supplementary Figure S3B. Data are presented as mean ± SEM, and the mean differences were analyzed using the two-way analysis of variance (ANOVA) followed by Tukey’s multiple comparison tests. *p < 0.05, **p < 0.01, ***p < 0.001 between NT and HS conditions within the treatment group. Different lowercase letters indicate significant differences between NT treatment groups, and uppercase letters indicate significant differences between HS treatment groups.

Gene expression analysis revealed that *NRF2* transcript levels were upregulated in all antioxidant-treated groups under HS conditions compared to the respective control, with statistical significance for only HS QUE (*p = 0.0260*) and HS SFN (*p = 0.0163*) compared to HS control (Supplementary Figure S3). Moreover, increased expression of downstream antioxidant genes *SOD1, HO1, and PRDX1 (p < 0.05)* was observed in the SFN-treated group compared with the nontreated control and vehicle groups under HS condition. Quantification of nuclear NRF2 protein in cultured GCs demonstrated an increase in QUE (*p = 0.0002*) and SFN (*p = 0.0007*) treated groups under HS conditions compared to the non-supplemented HS control and vehicle groups (Figure 1A). ROS accumulation was reduced for QUE (*p < 0.0001*), CAR (*p < 0.0001*), SFN (*p < 0.0001*), and MIX (*p < 0.0001*) compared to the control and vehicle under HS conditions (Figure 1B). Additionally, MMP was increased in GCs treated with QUE (*p = 0.0130*), CAR (*p = 0.0048*), SFN (*p = 0.0382*), and MIX (*p < 0.0001*) under conditions of HS compared to the control and vehicle-treated groups (Figure 1C), indicating improved mitochondrial function and maintenance of cellular homeostasis. Reductions in the total rate of apoptosis in GCs were also observed for QUE (*p = 0.0187*), CAR (*p = 0.0234*), SFN (*p = 0.0165*), and MIX (*p = 0.0228*) compared to the control group under HS conditions (Figure 1D; Supplementary Figure S3). Among the three antioxidants evaluated in cultured GCs, QUE and SFN induced a statistically significant increase in nuclear activation of the NRF2 protein, as determined by nuclear extract ELISA assays (Figure 1A). Therefore, given the central role of NRF2 in regulating antioxidant defense mechanisms, subsequent oocyte experiments were conducted using only these two antioxidants, QUE and SFN.

### 3.3 Antioxidant supplementation effectively mitigates the detrimental effects of HS during oocyte maturation via modulating the physiology of the surrounding CCs

**FIGURE 2:**
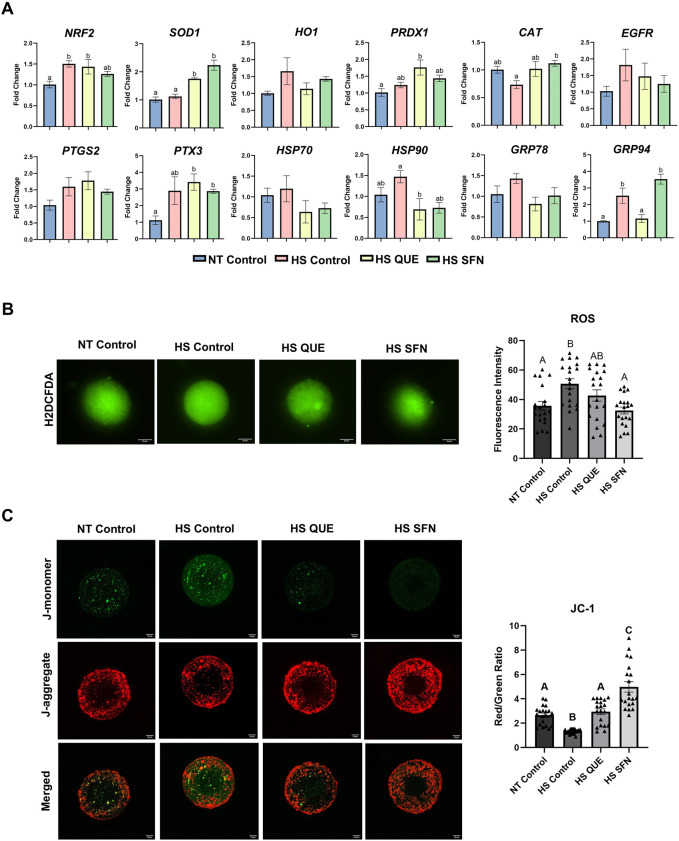
The protective effect of antioxidant supplementation mitigating HS effects on cumulus cells transcripts and oocyte function following maturation. **(A)** Stress-related gene expression analysis in cumulus cells, qRT-PCR was performed to quantify selected stress-related genes *NRF2, SOD1, HO1, CAT, PRDX1, HSP70, HSP90, GRP78, GRP94,* as well as cumulus cell expansion genes *PTGS2, EGFR*, and *PTX3.* Gene expression analysis in denuded oocytes did not show variations and is shown in Supplementary Figure S4. Results are from cumulus cells removed from four pools, each containing 50 oocytes, for a total of 200 oocytes per treatment. Data are presented as mean ± SEM, and the mean differences were analyzed using the One-way analysis of variance (ANOVA) followed by Tukey’s multiple comparison tests. Different lowercase letters indicate significant differences between treatment groups. **(B)** Reactive Oxygen Species (ROS) accumulation assay 2′,7′- DCFH-DA quantification of the relative fluorescence intensity of Reactive Oxygen Species (ROS) in single oocytes was carried out in triplicate (total = of 15–20 oocytes per treatment). Fluorescence images of *in vitro* matured bovine oocytes treated with H2DCFDA were captured for the measurement of intracellular ROS levels in single oocytes. Scale bar, 20 μM. **(C)** Fluorescence images of *in vitro* matured bovine oocytes treated with 5,5′,6,6′tetrachloro-1,1′,3,3′tetraethylbenzimidazolylcarbocynanine iodide (JC-1) for the measurement of MMP (δΨm), an indicator of mitochondrial activity in single oocytes. Scale bar, 20 μM. Quantification of the relative fluorescence intensity (red/green ratio) in single oocytes was carried out in duplicates (total = of 15–20 oocytes per treatment). Data are presented as mean ± SEM, and the mean differences were analyzed using the One-way analysis of variance (ANOVA) followed by Tukey’s multiple comparisons. Different upper case letters indicate a statistical difference between treatment groups p ≤ 0.05).

Cumulus cells displayed distinct transcriptional responses to antioxidant supplementation under HS conditions (Figure 2A). Our results showed that *HSP90* expression was reduced in the QUE-treated group compared to the HS control (*p = 0.04*), while *GRP94* expression was also lower in the QUE-treated group (*p = 0.008*). Similarly, *SOD1* expression was upregulated in CCs from both the QUE group (*p = 0.003*), and the SFN group (*p = 0.0001*). Also HS SFN showed increased expression of *CAT* (*p = 0.02*) compared to the HS control group. However, transcript levels in denuded oocytes remained unaltered across all treatment groups (Supplementary Figure S4). ROS accumulation assay revealed that both antioxidants successfully reduced ROS accumulation (Figure 2B). Consistently, the MMP assay assessed by JC-1 staining was enhanced in both the QUE (*p = 0.0004*) and SFN-treated (*p < 0.0001*) groups compared to the HS control group (Figure 2C).

### 3.4 The effect of antioxidant supplementation during oocyte maturation on the developmental capacity of oocytes following IVF

**FIGURE 3:**
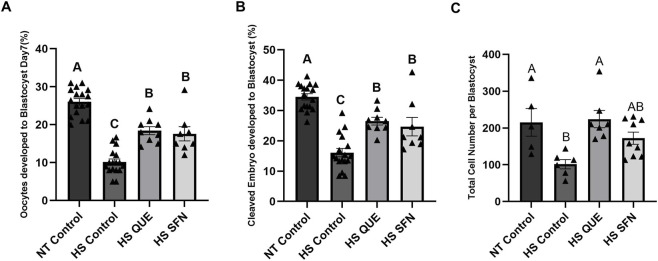
The effect of antioxidant supplementation on the developmental capacity of oocytes matured under Heat Stress. Results are from a total of 8-9 replicates (780–900 total oocytes per treatment). **(A)** Percentage of oocytes developed into blastocysts. **(B)** Percentage of cleaved embryos developed to blastocyst. **(C)** Total cell number per single blastocyst, quantification of total cell numbers in single blastocysts was carried out in quadruples (a total of 5–10 blastocysts per treatment). Data are presented as mean ± SEM, and the mean differences were analyzed using the One-way analysis of variance (ANOVA) followed by Tukey’s multiple comparisons. Different uppercase letters show a statistically significant differences between treatment groups with p ≤ 0.05.

As shown in Figure 3A, compared to those cultured under thermoneutral conditions, exposure of oocytes to HS results in a drastic reduction in the number of oocytes that develop to the blastocyst stage following IVF (*p < 0.0001*). However, priming oocytes with both QUE and SFN resulted in higher blastocyst rates compared to the non-supplemented HS group with *p < 0.0001* and *p = 0.0002,* respectively, following the same trend in blastocyst rate from cleaved embryos (Figure 3B). Similarly, while HS reduced total cell number per blastocyst compared to the NT group (*p = 0.0166*; Figure 3C), supplementation with QUE and SFN restored the cell number to the level comparable to the NT group.

### 3.5 Antioxidant supplementation under HS conditions during IVM modulates ROS accumulation and mitochondrial membrane potential of the resulting blastocysts

**FIGURE 4:**
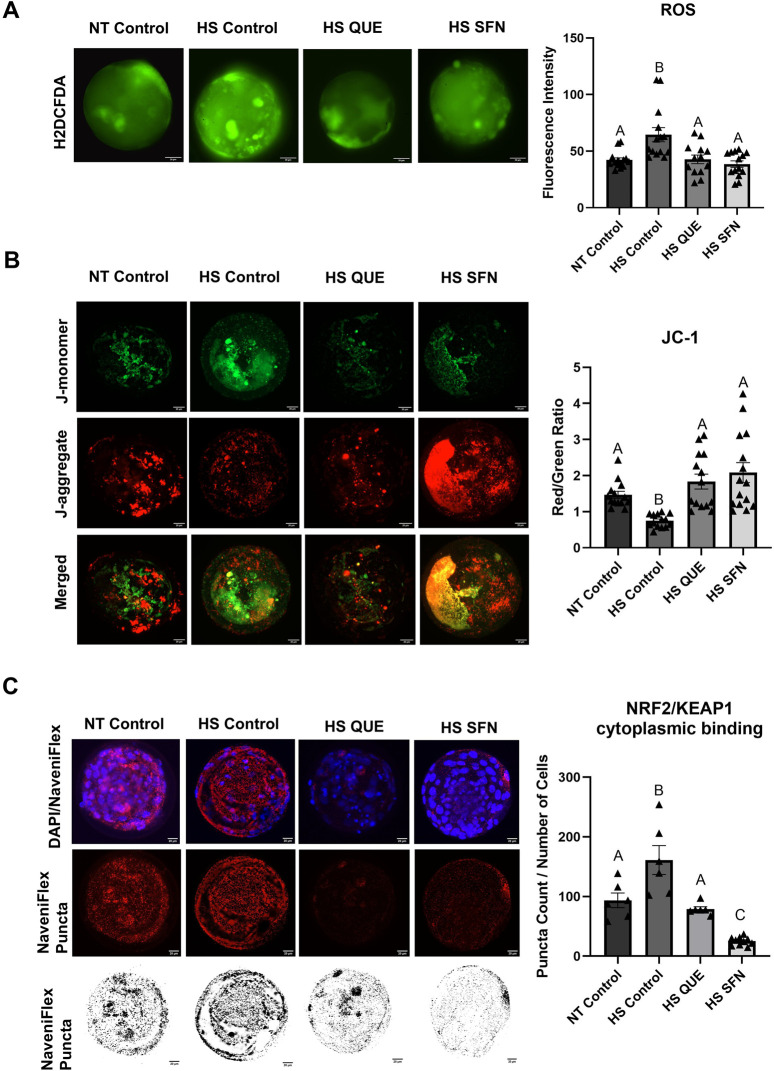
Functional alterations of antioxidant supplementation under HS conditions during *in vitro* maturation on blastocyst quality and NRF2-KEAP1 Protein-Protein Interaction. **(A)** Fluorescence images of *in vitro* cultured bovine blastocysts treated with 2ʹ,7ʹ-Dichlorofluorescin Diacetate (H2DCFDA) were captured for the measurement of intracellular ROS levels in single blastocysts. Scale bar, 20 μM. Quantification of the relative fluorescence intensity for reactive oxygen species (ROS) in single blastocysts was carried out in triplicate (a total of 12–15 blastocysts per treatment). **(B)** Fluorescence images of *in vitro* cultured bovine blastocysts treated 5,5′,6,6′tetrachloro-1,1′,3,3′tetraethylbenzimidazolylcarbocynanine iodide (JC-1) for the measurement of MMP (δΨm), an indicator of mitochondrial activity in single blastocysts. Scale bar, 20 μM. Quantification of the relative fluorescence intensity (red/green ratio) in single blastocysts was carried out in duplicates (a total of 12–15 blastocysts per treatment). **(C)** To further investigate the mechanistic role of antioxidant signaling in embryonic development, the colocalization of KEAP1 and NRF2 protein-protein interaction was evaluated using the highly sensitive Proximity Ligation Assay (PLA) and confocal fluorescence microscopy. Representative fluorescence images show Naviflex Pucta (red fluorescence) represents cytoplastic binding between NRF2 and KEAP1 in different treatment groups. Puncta count was optimized based on the total cell number of the corresponding embryo. A total of 6–10 individual embryos were used per group for four different replicates. Fewer puncta were correlated with greater free, active NRF2 availability in the blastocyst cytoplasm. Data are presented as mean ± SEM, and the mean differences were analyzed using the One-way analysis of variance (ANOVA) followed by Tukey’s multiple comparisons. Different uppercase letters show a statistically significant differences between treatment groups with p ≤ 0.05.

To elucidate the long-term impact of antioxidant priming and HS exposure during oocyte maturation on the molecular architecture and viability of the resulting blastocysts, various metabolic and molecular parameters were investigated. As shown in Figure 4A, although IVF and IVC were conducted under thermoneutral conditions without antioxidant supplementation, priming oocytes with QUE or SFN effectively restored ROS levels in blastocysts to those observed under thermoneutral conditions, compared with HS groups (HS QUE, *p = 0.0024*; HS SFN, *p = 0.0002*). Furthermore, both antioxidant treatments successfully recovered MMP of blastocysts compared with the non-supplemented HS control (HS QUE*, p = 0.0008*; HS SFN, *p < 0.0001*).

To further investigate the impact of antioxidant supplementation on the activity of the NRF2-KEAP1 signaling pathway in the resulting blastocysts, the highly sensitive Proximity Ligation Assay (PLA) was used to assess protein-protein interactions between KEAP1 and NRF2 (Figure 4C). In representative images, Naviflex Puncta (red fluorescence), the cytoplasmic binding between NRF2 and KEAP1 across different treatment groups was presented in puncta. Antioxidant supplementation during IVM promoted dissociation of KEAP1-NRF2 complexes, as evidenced by reduced puncta count per blastomere compared with the HS control group (HS QUE *p = 0.0007*; HS SFN *p < 0.0001*). Puncta counts were normalized to the total cell number of each corresponding embryo.

### 3.6 Metabolic function of preimplantation embryos derived from antioxidant primed oocytes matured under HS conditions

**FIGURE 5:**
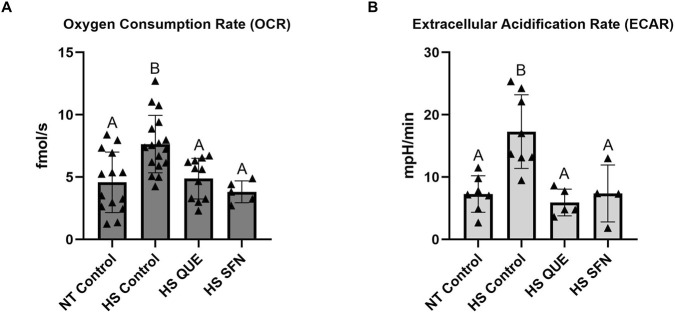
Metabolic function of preimplantation embryos from antioxidant-treated oocytes matured under HS conditions. The Metabolic multisensor platform was used on single pre-implantation embryos to quantify: **(A)** Oxygen Consumption Rate (OCR) in quercetin and sulforaphane treatments under HS conditions measured in single embryos (n = 4–17 per group). **(B)** Extracellular Acidification Rate (ECAR) in quercetin and sulforaphane treatments under HS conditions measured in single embryos (n = 4-7 per group). Data are presented as mean ± SEM, and the mean differences were analyzed using the One-way analysis of variance (ANOVA) followed by Tukey’s multiple comparisons. Different uppercase letters show a statistically significant differences between treatment groups with p ≤ 0.05.

In the present study, we integrated a metabolic multi-sensor platform capable of monitoring a single embryo oxygen consumption rate (OCR) and extracellular acidification rate (ECAR) in real-time, by applying amperometric methods described in (Obeidat Y. et al., 2019). The use of this microchamber enables monitoring of single-embryo metabolism during development in a small volume of media. OCR, which is an indicator of aerobic metabolism, was measured in fmol per second per embryo. ECAR, which is an indirect marker of anaerobic metabolism, was measured in mpH per minute per embryo. As shown in Figure 5, supplementation with both QUE and SFN reduced OCR to the thermoneutral level, compared with non-supplemented HS control groups (HS QUE, *p = 0.0078*; HS SFN, *p = 0.0048*). Similarly, ECAR values were reduced to levels comparable to the thermoneutral embryos when compared to the HS control group (HS QUE *p = 0.0009*; HS SFN *p = 0.0067*).

### 3.7 Antioxidant supplementation during oocyte maturation restored the developmental capacity of blastocysts as determined by embryo competence index

**FIGURE 6:**
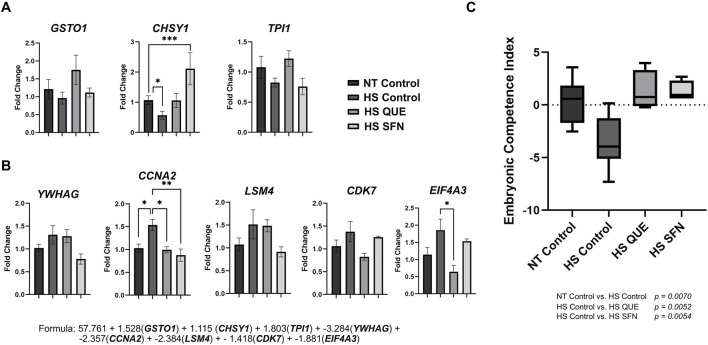
The Embryonic Competence Index (ECI) is restored by antioxidant supplementation during IVM under HS. This predictive tool is based on a Bayesian multiple regression formula that identifies transcriptomic biomarker abundance to distinguish in vivo-derived blastocysts that resulted in pregnancy from those that did not, and it validates a predictive formula for assessing embryo vitality. qRT-PCR was performed to quantify: **(A)** Markers corresponding to glutathione S-transferase omega 1 (*GSTO1*), chondroitin sulfate synthase 1 (*CHSY1*), and triosephosphate isomerase 1 (*TPI1*), which are increased in competent embryos. **(B)** Markers: tyrosine 3-monooxygenase/tryptophan 5-monooxygenase activation protein gamma (*YWHAG*), cyclin A2 (*CCNA2*), LSM4 homolog (U6 small nuclear RNA and mRNA degradation associated, *LSM4*), cyclin-dependent kinase 7 (*CDK7*), and eukaryotic translation initiation factor 4A3 (*EIF4A3*) increased in noncompetent embryos. **(C)** Embryonic Competence Index (ECI). The following formula (R2 = 0.96) was used to determine the ECI of blastocysts based on the expression level of the 8 candidate genes: ECI = 57.761 + 1.528(*GSTO1*) + 1.115 (*CHSY1*) + 1.803(*TPI1*) + −3.284(*YWHAG*) + −2.357(*CCNA2*) + - 2.384(*LSM4*) + −1.418(*CDK7*) + −1.881(*EIF4A3*). Data are presented as mean ± SEM, and the mean differences were analyzed using one-way analysis of variance (ANOVA) followed by Tukey’s multiple comparisons. Statistical differences for each gene between treatment groups is denoted with *p < 0.05, **p < 0.01, ***p < 0.001. P-values for ECI comparisons between treatment groups are shown in the graphic.

As shown in Figure 6, the ECI values were reduced for blastocysts derived from non-supplemented HS control oocytes compared with the thermoneutral group (*p = 0.0070*). However, supplementation with QUE and SFN during oocyte maturation under thermal stress conditions rescues the ECI of the resulting blastocysts compared with HS controls (*p = 0.0052* and p = *0.0054*, respectively). Among the competence-associated genes (Figure 6A), CHSY1 was significantly reduced in the non-supplemented HS control group compared to the thermoneutral control group (*p = 0.0339*). However, the supplementation of SFN increased CHSY1 expression compared to the HS control group (*p = 0.0008*). Among the non-competence-associated genes (Figure 6B), CCNA2 was upregulated in the HS control group compared to the NT control (*p = 0.0149*), while supplementation of QUE and SFN downregulated CCNA2 expression as compared to the HS control (*p = 0.0464* and 0.0047, respectively). Moreover, QUE supplementation reduced EIF4A3 expression compared to the HS control group (*p = 0.0180*). Collectively, supplementation with QUE and SFN during oocyte maturation under thermal stress conditions rescues the ECI of the resulting blastocysts compared with their non-treated thermal stressed counterparts (Figure 6C).

## 4 Discussion

Environmental thermal stress remains one of the most detrimental factors compromising reproductive efficiency in livestock, exerting profound effects on ovarian function, oocyte maturation, and early embryo development through oxidative stress, mitochondrial dysfunction, endoplasmic reticulum stress, and apoptosis (Amaral et al., 2020; Gaskins et al., 2021; Sammad et al., 2020). The objective of this study was therefore to determine whether supplementation with plant-derived antioxidants during *in vitro* culture of bovine follicular cells and oocytes under HS could mitigate oxidative and mitochondrial damage in GCs, oocytes, and restore the developmental competence of the resulting embryos.

As an initial screening step, we evaluated three antioxidants (QUE, CAR, and SFN), as well as their combination, in GCs exposed to HS, assessing cellular function, maintenance of oxidative homeostasis, and their ability to activate NRF2. Although all antioxidants improved redox homeostasis, only QUE and SFN induced a significant increase in nuclear NRF2 activation (Figure 1A), as confirmed by our nuclear extracts ELISA assays. Because NRF2 activation is a key regulator of antioxidant defense mechanism, these results provided a mechanistic rationale to narrow subsequent IVM experiments using QUE and SFN. This approach ensured that only compounds showing both functional improvement and mechanistic role in activating the antioxidant capacity of the oocytes and embryos via NRF2-KEAP1 pathway were selected.

Oocytes are particularly vulnerable to thermal and oxidative insults, with sensitivity reported as early as 105 days before ovulation and persisting through ovulation and fertilization (Torres-Júnior et al., 2008). Given this prolonged window of susceptibility, impaired developmental outcomes under HS conditions are largely attributable to compromised oocyte quality rather than sperm factors (Rahman et al., 2018; Roth, 2017). Excess ROS in the ovary deteriorates oocyte competence, induces GCs apoptosis, disrupts GCs–oocyte communication, alters cytoskeletal and spindle assembly dynamics, and accelerates ovarian aging through mitochondrial dysfunction, telomere shortening, inflammation, and apoptosis (Prasad et al., 2016; Yaacobi-Artzi et al., 2022; Yang et al., 2021). Consistent with these observations, HS in our model increased ROS and reduced MMP in oocytes (Figures 2B,C), despite minimal transcriptomic changes in the oocyte itself (Supplementary Figure S4). However, transcripts in the CCs exhibited clear antioxidant activation as both QUE and SFN increased NRF2 downstream genes (Figure 2A). ROS accumulation (Figure 2B), markedly elevated under HS, was reduced by both antioxidants to levels comparable to the NT control. The MMP, which is reduced under HS, as previously reported (Menjivar et al., 2023), was restored by QUE and SFN, demonstrating the ability of these antioxidants to preserve mitochondrial function during IVM. These findings parallel previous reports where SFN alleviated chemically induced oxidative stress in mouse oocytes (Li et al., 2023), and protected bovine oocytes against paraquat toxicity through NRF2-mediated mechanisms (Feng et al., 2023). The increased expression of NRF2-downstream genes in cumulus cells (Figure 2A) further supports the role of the cumulus compartment in mediating antioxidant protection during oocyte maturation. Together, our data reinforce that QUE and SFN offer a targeted strategy to counteract HS-induced oxidative stress during IVM via modulating the antioxidant capacity of the surrounding cumulus cells.

Thermal stress exposure during oocyte maturation induced a pronounced carryover effect, leading to reduced blastocyst development (Figures 3A,B), likely due to the cumulative impact of oxidative stress on embryonic developmental capacity. Moreover, the resulting embryos exhibited elevated ROS levels (Figure 4A) and decreased MMP (Figure 4B), consistent with our observation in GCs and oocytes. To evaluate whether antioxidant supplementation during IVM influenced NRF2 signaling in the blastocysts, we employed the highly sensitive protein-protein interaction assay, to assess KEAP1–NRF2 interactions (Figure 4C). In control embryos derived from non-supplemented heat-stressed oocytes, abundant KEAP1–NRF2 puncta indicated cytoplasmic sequestration of NRF2. In contrast, QUE and SFN supplementation during IVM significantly reduced puncta counts per blastomere, reflecting greater availability of free, active NRF2 in the cytoplasm (Figure 4C). These findings mirror our observations in GCs, where both antioxidants increased NRF2 translocation into the nucleus, despite exposure to thermal stress. This concept aligns with our previous studies showing that QUE during bovine embryo culture activates NRF2 and improves mitochondrial activity and developmental outcomes (Khadrawy et al., 2020).

On the other hand, HS exerts profound adverse effects on early embryo development, particularly prior to zygotic genome activation (ZGA), and is a major contributor to early pregnancy loss in cattle (de Barros and Paula-Lopes, 2018). Recent advances in embryo biology have identified molecular markers predictive of embryonic competence, enabling the development of the Embryonic Competence Index (ECI) to quantify the likelihood of embryo survival and pregnancy establishment (Rabaglino et al., 2023; Rabaglino and Hansen, 2024). This index is dependent on the expression of competence-associated genes (*GSTO1, CHSY1*, and *TPI1) involved in* energy metabolism, and non-competence-associated genes (*CCNA2, CDK7, EIF4A3, YWHAG*, and *LSM4*) involved in mRNA processing and cell cycle regulation. In the present study, we examined whether antioxidant supplementation (QUE and SFN) during IVM under HS conditions could restore embryonic competence, using both ECI assessment and single-embryo metabolic measurements (Figures 5A,B). Restoration of ECI using antioxidant supplementation was accompanied by reduced OCR and ECAR compared with HS controls, indicating that embryo competence is linked to the level of embryonic energy expenditure (Leese et al., 2022). Reduction of both OCR and ECAR to levels comparable to non–heat-stressed (NT) controls following antioxidant supplementation indicated stabilization of embryonic energy metabolism, consistent with the “Quiet Embryo Hypothesis” (Leese, 2002; Leese et al., 2022), which proposes that developmentally competent embryos exhibit lower metabolic activity than less viable counterparts. These results suggest that QUE and SFN not only stabilize metabolic activity but also positively modulate gene networks underlying embryo competence, enhancing the likelihood of sustaining pregnancy. By incorporating ECI into our study, we demonstrate that antioxidant supplementation during oocyte maturation can mitigate the long-term impact of thermal stress on embryos' ability to induce pregnancy after transfer.

Collectively, our findings demonstrate that supplementation of QUE and SFN during oocyte maturation under thermal stress conditions attenuates the detrimental impact of HS in oocytes, stabilizes energy metabolism, and restores embryonic competence by improving the antioxidant capacity of the oocytes and surrounding cumulus cells and the resulting blastocysts via activating NRF2 mediated oxidative stress response pathway. To our knowledge, this is the first report demonstrating that the antioxidants QUE and SFN can effectively reverse the long-term consequences of heat stress in oocytes, including impaired metabolic efficiency and altered embryonic competence in preimplantation embryos. These findings highlight a promising strategy to improve assisted reproductive technology (ART) outcomes under environmental stress conditions and provide a mechanistic foundation for integrating pharmacological approaches to enhance antioxidant capacity in ovarian cells, oocytes and preimplantation embryos in livestock species.

## Data Availability

The datasets presented in this study can be found in online repositories. The names of the repository/repositories and accession number(s) can be found in the article/Supplementary Material.

## Supplementary material

SUPPLEMENTARY FIGURE 1 Experimental models for **(A)** Bovine granulosa cell in vitro culture. **(B)** In vitro maturation. **(C)** In vitro embryo production. Antioxidant supplementation and heat stress exposure.

SUPPLEMENTARY FIGURE 2 Dose-dependent impact of antioxidant supplementation on bovine granulosa cell viability. Cell Viability assay CCK8 for each antioxidant under heat stress (HS) and normal temperature (NT). **p < 0.01; ***p < 0.001 between NT and HS conditions within the treatment group; different lowercase letters show significant differences between NT treatment groups and uppercase letters for HS treatment groups. Data are presented as mean ± SEM, and the mean differences were analyzed using the two-way analysis of variance (ANOVA) followed by Tukey’s multiple comparison tests.

SUPPLEMENTARY FIGURE 3 The impact of antioxidant supplementation on stress-related genes and apoptotic rate. **(A)** qRT-PCR was performed to quantify selected stress-related genes NRF2, SOD1, HO1, PRDX1, CAT, HSP70, HSP90, GRP78, and GRP94. Data are presented as mean ± SEM, and the mean differences were analyzed using the two-way analysis of variance (ANOVA) followed by Tukey’s multiple comparison tests. *p < 0.05, **p < 0.01, ***p < 0.001 between normal temperature (NT) and heat stress (HS) conditions within the treatment group. Different lowercase letters indicate significant differences between treatment groups under NT, and uppercase letters indicate significant differences between treatment groups under HS condition. **(B)** Representative panels show the distribution of cells stained for both Annexin V and PI across the different quadrants between the different treatment groups. Blue bars represent Normal Temperature groups, and red bars represent Heat Stress treatments.

SUPPLEMENTARY FIGURE 4 The impact of antioxidant supplementation on transcript levels of stress-associated genes in denuded oocytes following maturation. To assess stress-related gene expression in denuded oocytes, qRT-PCR was performed to quantify *NRF2, SOD1, HO1, CAT, PRDX1, HSP70, HSP90, GRP78,* and *GRP94*. Results were obtained from denuded oocytes from four replicates (a total of 200 denuded oocytes per treatment). Stress-related gene expression analysis in cumulus cells, qRT-PCR was performed to quantify selected stress-related genes *NRF2, SOD1, HO1, CAT, PRDX1, HSP70, HSP90, GRP78, GRP94*, as well as cumulus cell expansion genes *PTGS2, EGFR*, and *PTX3*. Results are from cumulus cells removed from a total of four pools, each containing 50 oocytes, for a total of 200 oocytes per treatment. Data are presented as mean ± SEM, and the mean differences were analyzed using the One-way analysis of variance (ANOVA) followed by Tukey’s multiple comparison tests. Different lowercase letters indicate statistically significant differences between treatment groups.
