# Supplementary figures and images for: Enhancing antioxidant capacity via NRF2 pathway activation to mitigate heat stress‐induced oxidative damage in bovine granulosa cells, oocytes, and embryos
Source: Front Cell Dev Biol. 2026 Feb 12;14:1777760. doi: 10.3389/fcell.2026.1777760 (PMC12936007; doi:10.3389/fcell.2026.1777760)

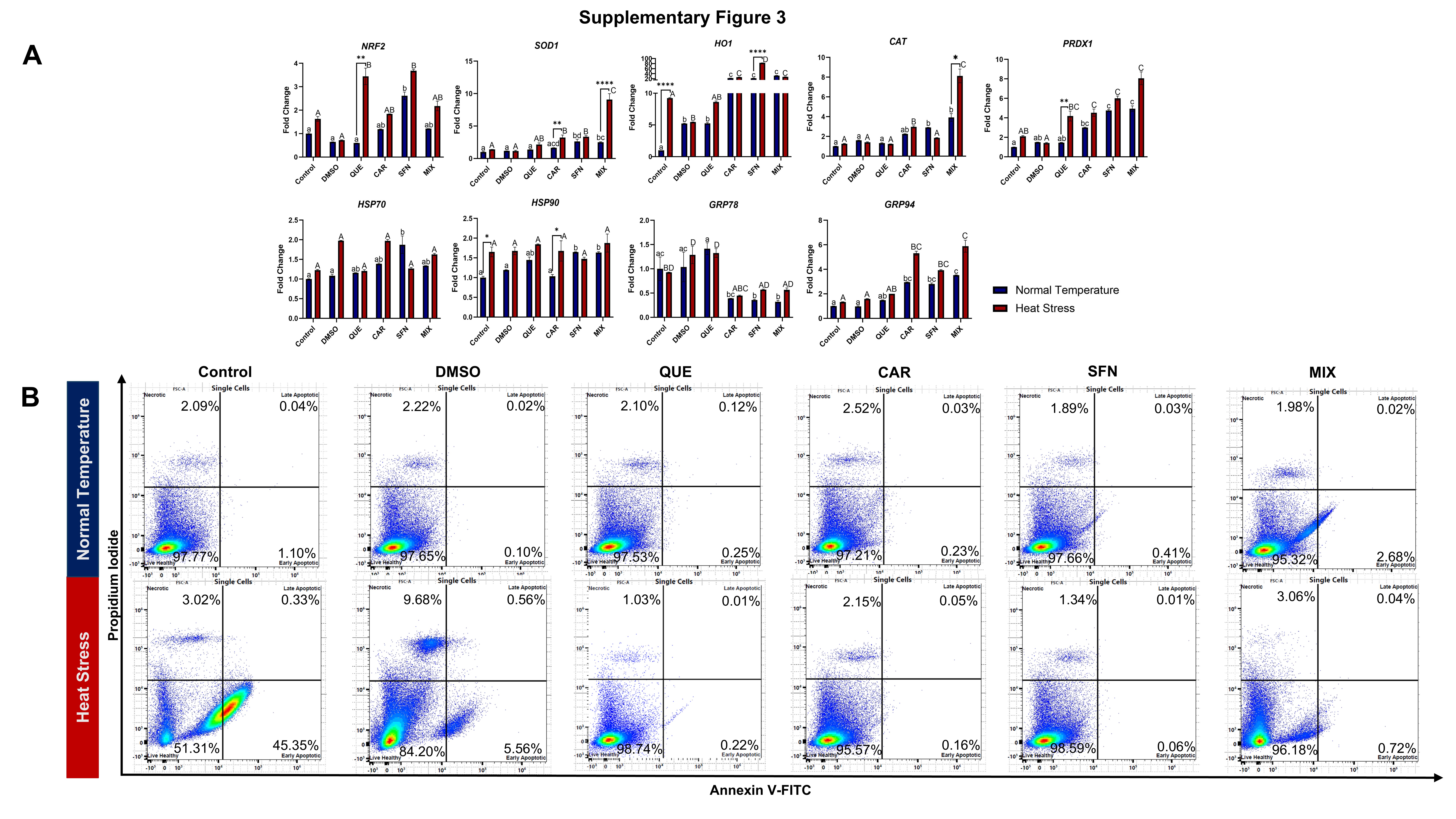

Supplement: Supplementary file 1 [file Image3.jpeg]

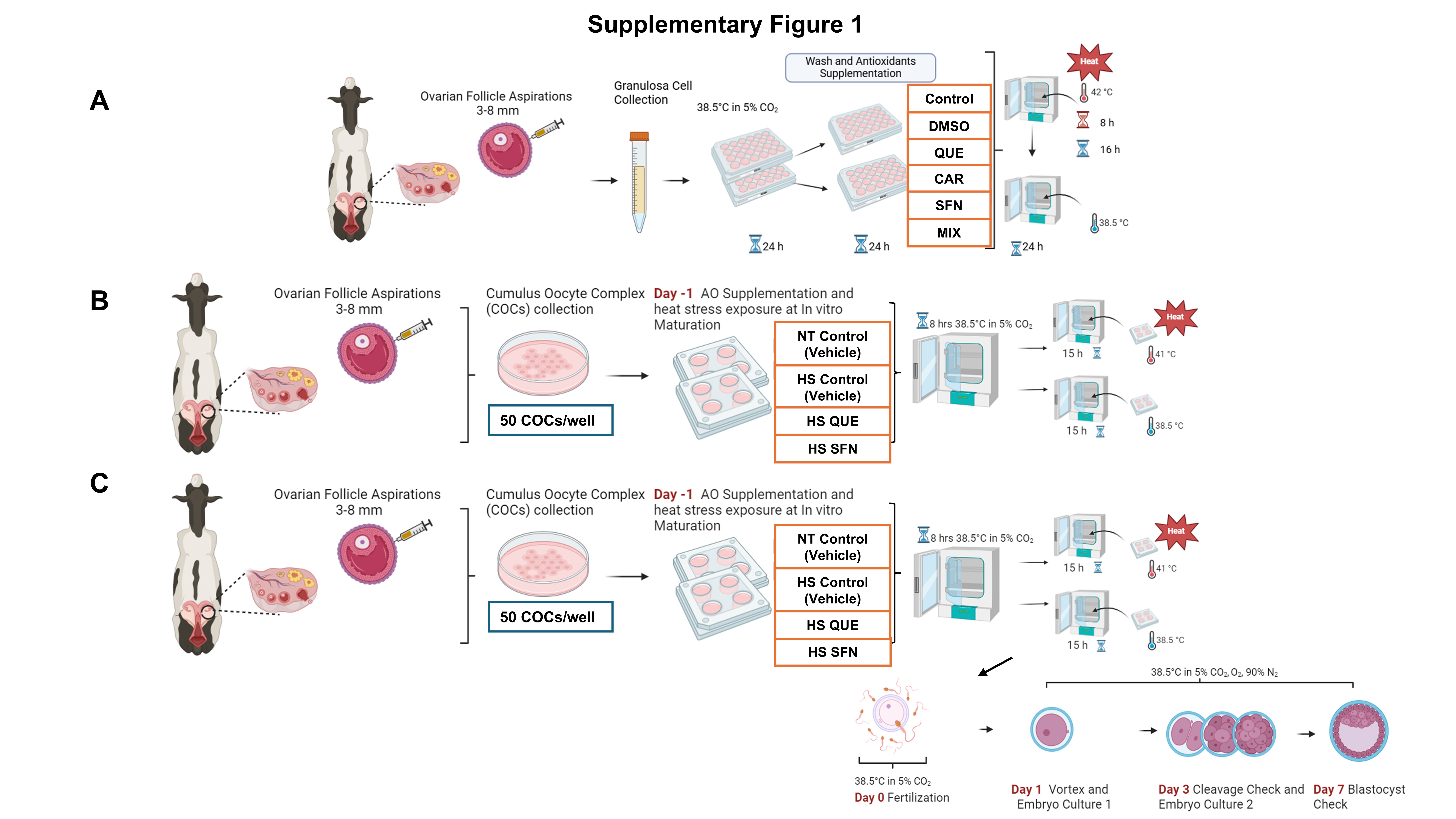

Supplement: Supplementary file 3 [file Image1.jpeg]

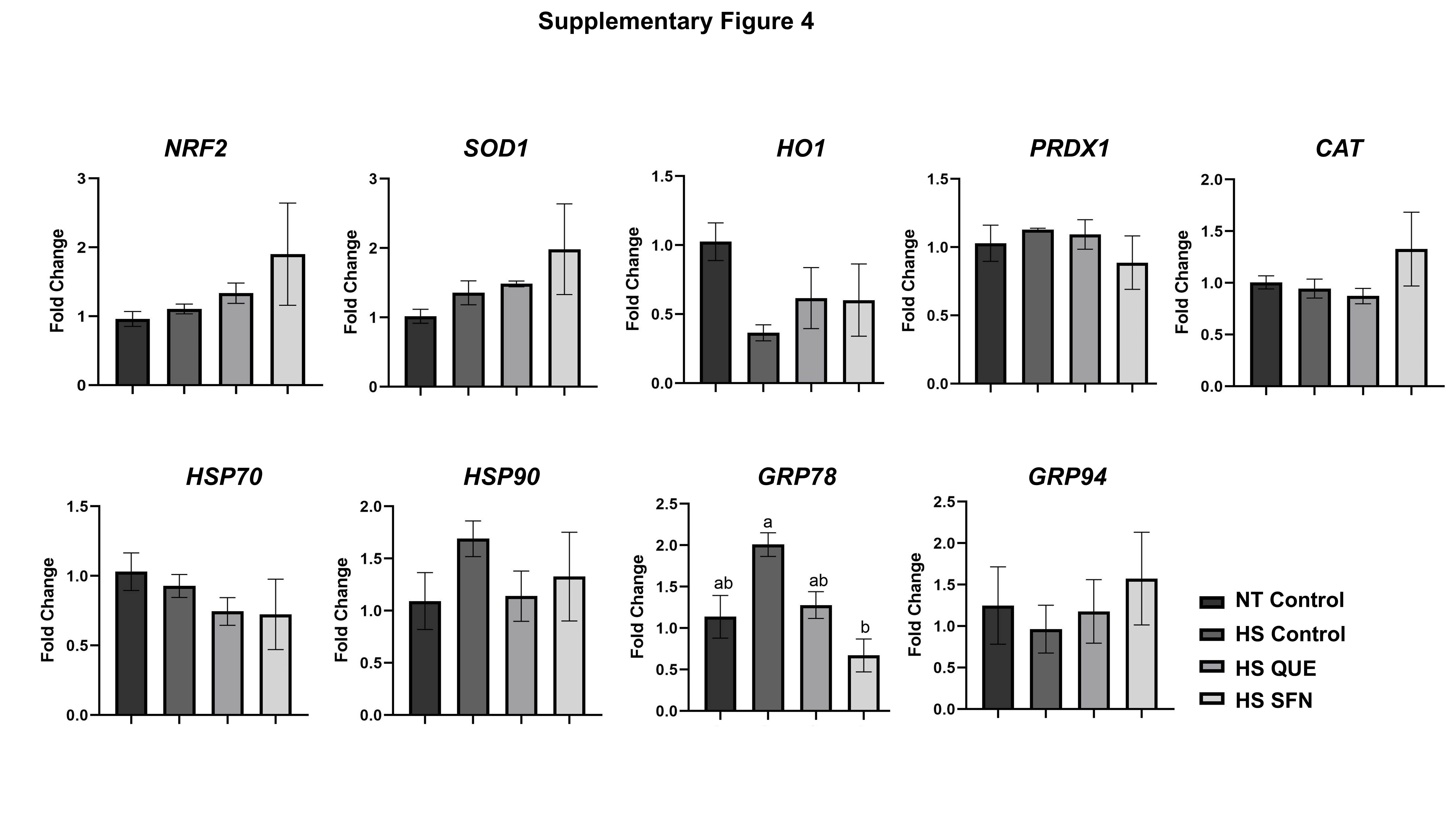

Supplement: Supplementary file 4 [file Image4.jpeg]

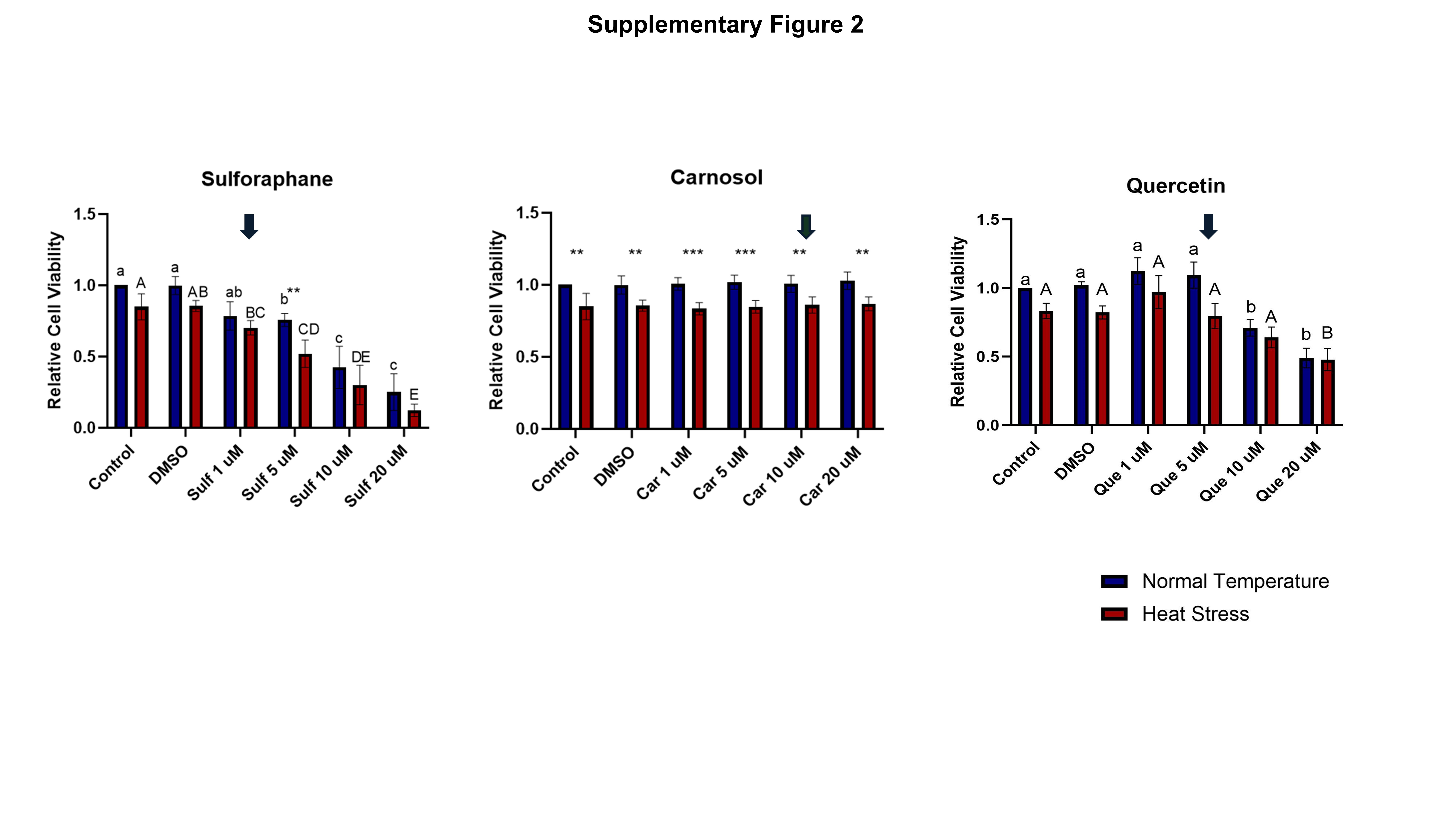

Supplement: Supplementary file 5 [file Image2.jpeg]
