# Supplementary material for: Enhancing antioxidant capacity via NRF2 pathway activation to mitigate heat stress‐induced oxidative damage in bovine granulosa cells, oocytes, and embryos
Source: Front Cell Dev Biol. 2026 Feb 12;14:1777760. doi: 10.3389/fcell.2026.1777760 (PMC12936007; doi:10.3389/fcell.2026.1777760)
Supplement: Supplementary file 2 [file Table1.docx]

| **Gene name** | **Accession number** | **Primer sequences** |
| --- | --- | --- |
| PTGS2 | NM_174445 | F5`-cgatgagcagttgttccaga-3´  R5´-gaaagacgtcaggcagaagg-3´ |
| EGFR | XM_002696890 | F5`-gacccgaaagaactggacat-3´  R5´-tgttatatccaggccgacaa-3´ |
| PTX3 | NM_001076259 | F5`-acctgggattcaaagaaagg-3´  R5´-caccctcccagatattgaag-3´ |
| NRF2 | NM_001011678 | F5´-cccagtcttcactgctcctc-3´  R5´-tcagccagcttgtcattttg-3´ |
| SOD1 | NM_174615 | F5´-agaggcatgttggagacctg-3´  R5´-cagcgttgccagtctttgta-3´ |
| HSP70 | NM_001038505 | F5´-aatgccagttgccaatgctg-3´  R5´-atcgagagttcctccaccca-3´ |
| HSP90 | NM_001012670 | F5´-tcactgaggaaatgccaccc-3´  R5´-atggagacagagcgctgaac-3´ |
| GRP78 | NM_001075148 | F5´-tgcgaagccctatagctgac-3´  R5´-agtaggtggtacccaggtcg-3´ |
| GRP94 | NM_174700 | F5´-tgctgtgtggagagggaatg-3´  R5´-tcctgtgaccacaatcccaa-3´ |
| β-ACTIN | NM_173979 | F5´-tgtccaccttccagcagat-3´  R5´-tcaccttcaccgttccagt-3´ |
| GAPDH | NM_001034034 | F5´-aatggagccatcaccatc-3´  R5´-gtggttcacgcccatcaca-3´ |
| CHSY1 | NM_001191157.1 | F5´-catggcgaggcccaggat-3´  R5´-tggaccacgtcctgaaggc-3´ |
| EIF4A3 | NM_001046188.2 | F5´-tttgtggctgtggaacggga-3´  R5´-gtcagccagtcaaccttcctct-3´ |
| CDK7 | NM_001075715.1 | F5´-aggccttggaacaaggaggatt-3´  R5´-tattgtgcctttttcactgtttcct-3´ |
| GSTO1 | NM_001075214.2 | F5´-accccggtgccagctttatc-3´  R5´-tgttgatgacttcatgccgga-3´ |
| TPI1 | NM_001013589.3 | F5´-ccgcatcatttatgggggttct-3´  R5´-agctacctagtccctggctca-3´ |
| YWHAG | NM_174793.2 | F5´-gccccggatcatcctcgtcc-3´  R5´-ttcagctccgtcacgttcttca-3´ |
| CCNA2 | NM_001075123.2 | F5´-taagacgcgacgggttgcac-3´  R5´-ggccaagacatcttcactttctga-3´ |
| LSM4 | NM_001035436.2 | F5´-gccgggcgaggtgtgttc-3´  R5´-tcgtctcgggaatggaggga-3´ |
| HMOX1 | NM_001014912.1 | F5´-gcaaggtgcaagacttggct-3´  R5´-cacatggcgtaaagccccac-3´ |
| PRDX1 | NM_174431.1 | F5´-tcctatttcagtggaactgat-3´  R5´-aagcaatgatctccgtgggg-3´ |
| CAT | NM_001035386.2 | F5´-tgggacccaactacttccag-3´  R5´-aagtgggtcctgtgttccag-3´ |
